# Simulated Interviews, Real Results: A feasibility Study Implementing “Standardized Research Participants” cases in Qualitative Research Training with Older Adults

**DOI:** 10.21203/rs.3.rs-6573498/v1

**Published:** 2025-07-07

**Authors:** Ignacia Arteaga, Alma Hernandez de Jesus, Brandi Ginn, Corey Abramson, Daniel Dohan

**Affiliations:** University of California, San Francisco; University of California, San Francisco; University of California, San Francisco; Rice University; University of California, San Francisco

**Keywords:** standardized patients, standardized research participants, research training, simulation laboratories, qualitative interview, novice researchers

## Abstract

Qualitative interview training often relies on informal approaches such as peer role-playing or experiential learning, with few structured methods available. Yet balancing the demand for high-quality data with the ethical imperative to prevent harm and minimize emotional distress is critical, particularly when engaging vulnerable populations such as older adults experiencing cognitive decline. We piloted a structured training program adapting simulated interview methods from clinical education. Standardized research participant cases were developed in collaboration with simulation specialists and older adult actors, and used in interviews conducted by novice staff researchers. Experienced investigators observed remotely, followed by structured debriefing and self-reflection. Staff reported increased confidence, improved interviewing skills, and enhanced awareness of participant well-being. Actors provided feedback on communication, rapport, and interview flow. This study demonstrates the feasibility and value of structured simulation-based training to improve qualitative research skills across a range of settings.

## INTRODUCTION

In-depth semi-structured interviews provide data about subjects’ perceptions, attitudes, and perspectives. They are a mainstay of qualitative research and appear in countless scholarly studies and publications. Interviews can generate rich and nuanced accounts of subjective experience, but the value and reliability of interview data can be influenced by interviewers’ ability to establish and maintain rapport, balance conversational flows and silences, and obtain study-relevant data within limited time.^1,2^ Skilled interviewers are able to interpret participants’ statements, probe appropriately for further detail, and move through interview topics while conveying a stance of engagement and active listening via tone of voice and body language.^3–5^ Qualitative researchers typically find that their ability to interview effectively improves with time and experience.^1^ Interviews conducted by novice or inexperienced researchers may be sub-optimal both in the quality of data they collect as well as in the interview experience for both research participant and researcher.^5,6^ Providing opportunities for novice and less-experienced researchers to develop their in-depth semi-structured interviewing skills could improve qualitative data collection.

Current teaching methods in qualitative research often utilize both peer-to-peer role playing,^7–12^ and in-the-field immersion to develop interviewing skills.^7,10–13^ Peer interviews typically involve one student interviewing another student in a methods class. This is logistically convenient, but peer interviews do not provide an opportunity to practice the skills that researchers need to navigate the challenges of in-depth semi-structured interviews in real life. This is especially the case for researchers or research projects that are seeking to understand populations and situations different from students in higher education, e.g. adults with few years of education or older adults.^14^ Field immersion with potential subjects outside the classroom offers a more realistic opportunity to practice interviewing skills but can raise logistical, safety and ethical concerns including the need for human subjects research protections.^5,10^ In any interviewer training, guidance from experienced researchers, instructor evaluations, and self-reflection are crucial components for identifying areas of improvement, and the learning curve is steep.

In the clinical health sciences, practitioners rely on in-depth semi-structured interviews with patients to obtain information critical for healthcare decision-making, and these fields have developed different methods to teach and assess student interview skills. One method is or students to practice by interviewing standardized patients, who are trained and employed in simulation laboratories,^15^ and who evaluate student performance.^16^ Standardized patients have become particularly important in U.S. medical education in the last decades as its curriculum shifted from knowledge-based education to performance-based assessment. Further galvanizing training with standardized patients, in 2004 the National Board of Medical Examiners made clinical skills a component of licensing requirements.^17^ Standardized patient training now both fulfills training requirements while efficiently and reliably advancing clinical enskillment by eliminating variability through highly-scripted encounters with standardized patients, reducing staff burden by reducing the need for training supervision by highly-experienced clinicians, and minimizing harm by training students before they encounter actual patients.^17^

Standardized patients are actors, often non-professionals and sometimes patients, who are trained to present a set of symptoms in a standardized fashion as they interact with students and trainees in a simulated clinic environment.^15^ Standardized patient training can address educational goals beyond basic clinical reasoning including informing students about disabilities^18,19^, understanding the impact of the social determinants of health,^20^, developing interprofessional dynamics in collaborative care,^21^ fostering a sense of professional identity,^22^ and assessing quality of clinical care in low- and middle-income countries.^16,23^

The flexibility and adaptability of standardized patient education makes it potentially useful for uses in education, training, and skills in non-clinical disciplines as well. Boutain & Hitti adapted a standardized patient simulation lab infrastructure to train multiple interviewers to perform a structured qualitative interview. They reported that training helped familiarize interviewers with protocols, improved verbal and non-verbal communication skills, increased comfort when addressing sensitive topics, and improved overall interview flow.^24^

In this paper, we describe a pilot project in which simulation lab infrastructure was used to train novice interviewers to conduct in-depth semi-structured interviews using a standardized research participant (SRP) approach. The SRP pilot aimed to prepare novice social science researchers to enter the field for a comparative ethnographic project of aging and cognitive decline among older adults.

## METHODS

A Standardized Research Participant (SRP) is similar to the clinically-oriented Standardized Patient role performed by actors in simulation laboratories, with the difference that in SRP cases, clinical details are reduced, details about participants’ social context are expanded, and SRP have wider latitude to respond to the interviewer in a spontaneous and unscripted fashion.

**Figure 1:**
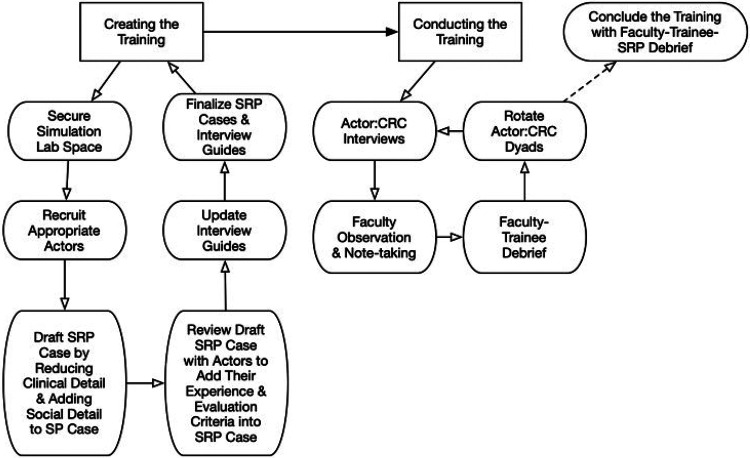
Key stages of the training protocol.

Figure 1 outlines the key stages of the training protocol piloted in this study. The process unfolded in two main phases: (1) designing the training scenario and materials, and (2) conducting the structured interview training with novice research staff. The first phase involved drafting and refining standardized research participant (SRP) cases in collaboration with older adult actors and simulation specialists. The second phase included rotating actor–staff interview sessions, structured faculty observation, and debrief opportunities between interview cycles. The training concluded with an open-ended debrief involving staff in training, actors, and faculty. This final discussion, which surfaced experiential insights and reflections, forms the primary data analyzed in this manuscript.

### Participants

The research team included two faculty members with qualitative research expertise (DD, CA) and four trainees (including AHdJ). The trainees were bachelor-level clinical research coordinators new to qualitative social science research who had been hired to conduct ethnographic fieldwork for an NIH-funded study examining experiences of aging and cognitive decline in the community. The pilot also engaged the skills and knowledge of the Kanbar Team, integrated by the operations manager at the Kanbar Center for Simulation and Clinical Skills at the University of California San Francisco (UCSF) and four actors as standardized research participants (SRP).

### Materials: Interview Guides and SRP Cases

The research team developed two semi-structured interview guides that reflected project research aims.^3,6^ One guide was to be used during interviews with older adults who were experiencing cognitive decline, and the other guide for interviews with caregivers of older adults experiencing cognitive decline. The guides included open-ended questions organized into discrete topic areas and included both stem questions to introduce a topic area and follow-up probes to explore respondents’ answers in greater depth.^5^ The guides presented interview themes in an organized logical progression, but trainees were expected to deviate from this order to establish rapport and maintain a conversational style as they responded to the narrative flow established by participants^4^.

In the health sciences, actors receive detailed case information to realistically portray a simulated patient. Standardized patient case templates include a general profile of the simulated patient, information about their appearance and emotional tone, historical and contextual information concerning physical and mental health, the patient’s physical condition, and the specific concern that brought them to clinic. The standardized patient case also includes visit-specific information such as how the patient should introduce themselves and respond to specific questions the case designers anticipate may arise during the clinical interview. Finally, the standard patient cases include criteria the actor should use to evaluate the performance of the student or trainee.

The Kanbar manager identified three standardized patient cases involving older adults to provide a starting point for developing the standardized research participant (SRP) cases for the pilot study: a person diagnosed with aphasia, a person with undiagnosed cognitive impairment, and a caregiver of a person living with dementia. The research team then adapted the standardized patient case to create the SRP cases by omitting or shortening sections of the original case such as clinical details irrelevant in a social science interview. The team also revised the case so that it reflected the context and tone of an open-ended research interview rather than a clinic-based medical exam by guiding actors that longer answers in a more conversational study were desirable. The SRP case asked actors to evaluate trainees performance based on the trainees’ success at introducing and explaining the study, putting them at ease, maintaining a natural flow of conversation, and eliciting study-relevant information^1^.

The research team met with the Kanbar team to review, revise, and edit the draft SRP cases. Based on Kanbar team feedback, we added further details about the SRP to foster a more realistic portrayal of the research participant and revised the case instructions to underscore that actors’ should draw on their own lived experience to make the SRP richer and more authentic. The aphasia SRP case was reviewed by a retired Standardized Patient actor recently diagnosed with aphasia. The finalized SRP cases thus included specific guidance about how a social science research subject would behave during a research interview as well as guidance to improvise during the interview based on personal lived experiences to ensure the interview felt authentic and spontaneous.

### Procedures: Simulated Interviews and Debriefing

Three experienced older adults who had been previously involved in simulations as standardized patients participated in the training by role-playing as an SRP. None had previously participated in non-clinical simulation and assessments. We provided each with an SRP case prior to the simulation session. The simulation session occurred during the course of a full day at the UCSF Kanbar center. The team used three interview rooms simultaneously. Every simulation room was wired to facilitate audio-visual recording and real-time monitoring from the Kanbar viewing center.

Each clinical research coordinator (trainee) interviewed an SRP for approximately one hour, and each trainee completed two interviews during the course of the simulation day. During the interview, the trainee explained the purpose of the study to the SRP, invited participation, gained informed consent, and conducted the substantive interview with support from the interview guide. The SRP agreed to be interviewed and responded to the substantive interview questions according to the SRP case characteristics provided beforehand. Senior project faculty monitored the interviews in real time from the viewing center. At the conclusion of the interview, the trainee, SRP, and faculty observers gathered to debrief and provide feedback. A second round of interviews followed immediately with each trainee interviewing a different SRP.

During the debrief, discussion topics included overall impressions, rapport building, comfort levels, communication styles, flow of conversation, body language, consenting research subjects, opening and closing interviews, how to engage in potentially sensitive subject material, similarity and difference from clinical interviews, and clarity and depth of questions.

## RESULTS

Resultant learnings emerged during the creation of the training, the simulation day, and subsequent debrief among the research team and trainees.

### Learning 1: Creating the Simulation Lab Training Exercise – transforming a Standardized Patient into a Standardized Research Participant (SRP).

Developing the SRP case involved more time and effort than expected. It provided unanticipated insights into the interviewing process, and it led to valuable opportunities for reflexivity. One source of unanticipated insight occurred at the first meeting with the older adult actors. The research team explained that during classroom-based role play interviews and field-based pilot interviews, individuals portraying “research participants” draw on their own life experiences and imagination to guide their performance in the role. The older adult actors recruited by the simulation lab manager balked at this approach for three reasons. First, it did not provide the actors with sufficient information to realistically portray their role. They needed more information about the nature of the cognitive decline they were experiencing as older adults or about the nature of the individual for whom they were providing care, and the kinds of caregiving work they were carrying out. Second, they felt that if they simply improvised during interactions, the trainees would not have the opportunity to interact in a standardized way. In their experience, establishing a standardized role was essential to ensure that the trainees had the opportunity to interact in a predictable fashion in the simulation lab encounters. Finally, they expressed concern about their ability to evaluate and provide feedback to the trainees and research team if they improvised the role. They felt this would impair their ability to focus on the learning objectives of the exercise and could be unfair to the trainees who would not know how their progress would be assessed or measured.

The actors asked specifically for guidance about their characters’ health and social background, how they should orient themselves to the research and behave interactionally, and what they should be looking for from the trainees as they assessed their performance and identified areas for improvement. To provide the actors with more guidance and information, the research team revised the standardized patient case template to create a standardized research participant (SRP) case. During this process, the research team reflected on the goals of the training and the research project. Trainees on the research team sought support with how to open the interview and navigate the required informed consent procedures while building a relationship with the participant. They also expressed a desire to train on how to use the interview guide with flexibility, e.g. to move between interview stems and probes and avoid reading one question after another. The team’s discussion of how to guide the actors led to a consideration of who should be considered an appropriate participant in the study, e.g. for older adult participants, what degree of cognitive impairment would make a participant appropriate or inappropriate for the study.

The research team drafted and reviewed SRP cases with the actors to obtain feedback about the realism and constraints of the case profiles. Researchers shared the purpose of the study with the actors and reviewed the significant differences between a clinical standardized patient case compared to our SRP case developed for a qualitative interview setting. Actors provided feedback about the case profiles – including feedback from the standardized patient actor who lives with aphasia about how his illness impacts his life.

Table 1 illustrates how SRP cases retain the rigor and intentionality of standardized patient design while adapting key elements to suit the goals of qualitative research training for an ethnographic project on experiences of aging and cognitive decline among older adults. Rather than standardizing pathology, SRP cases standardize context-rich life situations, allowing learners to practice empathetic, flexible, and grounded interviewing techniques.

### Learning 2: Simulation Lab Training Exercise

The simulation lab training involved two rounds of interviews per trainee that were observed by the faculty from the simulation lab control room. Trainees used study informed consent materials and its interview guide to interview each SRP. Following each interview, faculty and trainees met to debrief, and at the end of the interview session the research team debriefed with the SRP. Following the interview session, the trainees also had the opportunity to review videotapes of their interviews to facilitate further reflection.

After the first round of simulated interviews, faculty and trainees discussed the challenges and successes that trainees experienced at each stage of the interview. Table 2 summarizes the key learnings from research trainees about different interview stages. Trainees reported some degree of nervousness while conducting the interview although faculty (and later SRP) reported that their nervousness was not apparent. During discussion of the overall interview experience, faculty and trainees identified three arenas in which they wanted to focus their efforts at improvement. First, they sought to more quickly and naturally establish rapport to solicit detailed answers as the interview began. Second, they wanted to improve their ability to maintain a logical and coherent flow of conversation while simultaneously ensuring that they asked questions of relevance to the project’s research goals. Finally, they recognized a need to better anticipate and manage their response to emotionally charge or triggering topics.

During the additional round of interviewing, the trainees focused on improving their comfort at all stages of the interview. During research team debriefs after the second and third round of interviews, faculty and trainees felt that the interview entrance, greeting, and project introduction quickly improved. Trainees also made progress in carrying out the informed consent, audio recording, gift card, and closing stages while maintaining rapport with the SRP. The simulated interviews helped trainees identify how they would update the interview guide to address the challenge of maintaining a natural flow of conversation while covering research topics. Trainees watched each other’s video-recorded interviews, which enabled peer-to-peer feedback and discussion of how to approach different stages of the interview and problem-solve common challenges.

Once the practice interviews with SRPs were complete, the research team debriefed with the actors. The SRP feedback affirmed that there had been improvement during the simulation setting in several areas. The SRP appreciated the trainees’ focus on maintaining rapport and keeping the interview on a conversational level. The research team and SRP both noted that when SRP were able to draw on lived experience, the resultant interview felt richer to both parties. The SRP commended the trainees for how they approached emotional situations, noting their use of appropriate silence and consolation. However, the SRP also noted the need for continued improvement in how the trainees posed questions, particularly in avoiding jumbled or unclear questions and in improving their ability to shift smoothly from one interview topic to the next.

## DISCUSSION

An effective qualitative social science research interview gathers research-relevant data in field settings that may be unfamiliar to investigators and outside their control.^12,25^ Successful interviewers find creative ways to build rapport with participants, e.g., through self-disclosure and emotional attachment, without exhausting themselves emotionally.^26^ Ethnographic interviewing is thus a craft that builds on researchers’ experience and judgment not a set of procedures that can be taught and mastered once and for all. Learning to interview often occurs in classrooms via role-plays and during early fieldwork experiences via trial and error.

During the COVID-19 pandemic, restrictions on in-person contact made field-based interviewing difficult to conduct, particularly with vulnerable populations. This context created an opportunity to explore the feasibility and utility of using a simulation laboratory setting to train novice qualitative researchers. While simulation labs are a routine part of clinical education, they have not been widely applied in social science research training. By adapting the standardized patient model into a standardized research participant (SRP) approach, we introduced a structured, rigorous and replicable method for developing qualitative interviewing skills.

Similarly to standardized patient approaches in clinical training, the simulation lab allowed trainees to practice interview skills with realistic cases in a controlled environment, offering a closer approximation to field conditions than classroom-based role-plays. It also gave trainees the opportunity to engage with individuals likely to differ significantly from their own backgrounds—such as older adults or people living with serious illness. Given the vulnerability of the populations this project aimed to engage, the SRP approach provided a lower-risk, supportive setting for feedback and reflection, avoiding the ethical and logistical challenges of piloting interviews directly in the field. Thus, the SRP approach helped researchers gain familiarity with the technical, interpersonal, ethical, and emotional dimensions of in-depth interviewing^1^.

While simulation training has gained prominence in clinical education, scholars have raised important critiques that warrant attention. Anthropologist Janelle Taylor notes that standardized patient performances navigate a morally ambiguous space: they aim to portray suffering realistically without inflicting harm –what she refers to as both ‘real and not real’, yet rely on the emotional labor of real people working under precarious conditions and conceived as a ‘technology’ rather than a collaborator by most medical schools. She cautions that such simulations can teach to-be medical practitioners to perform caring behaviors for assessment purposes, without fostering deeper forms of ethical engagement.^17^

Our work qualitative research training with standardized research participants addressed these tensions to some degree. Rather than reproducing clinical simulations, we foregrounded narrative ambiguity, emotional complexity and self-awareness, aiming to cultivate ethical reflexivity as a foundational skill in qualitative interviewing. These commitments shaped not only how we structured the simulation, e.g, by inviting SRPs to give become active collaborators in the creation of the participant cases they portrayed, but also how faculty guided research team debriefs to support learning in interviewing participants who may be considered vulnerable by the nature of their cognitive decline.

A key insight of this pilot training exercise was that transforming standardized patient cases into standardized research participant (SRP) cases required more than simply removing clinical details. The adaptation process posed the challenge of developing cases that were socially meaningful and relevant within the broader context of the project’s ethnographic aims. Addressing this challenge became a learning opportunity. Through iterative collaboration with the actors, the research team refined each SRP’s identity and narrative, clarified the goals of the simulation, and identified the specific interviewing skills SRPs would observe and assess. In doing so, the team reflected more deeply on the kinds of participants they expected to encounter in the field and on what defined a successful interview for the project.

A second key insight was the recognition that how the interviewer introduces themselves and the study—and how they guide the participant through the informed consent process—has a lasting impact on the interviewer-participant relationship and the degree of rapport they can achieve during their encounter. Rather than treating consent as a procedural hurdle to overcome before the “real” interview begins, the training emphasized the importance of using these early moments to build connection and trust. This marked a shift from approaching consent as a legal requirement to be met using a formal, pre-specified script without deviation. That approach disrupted the flow of conversation before it could even begin and thus could irretrievably undermine relational dynamics. Framing consent as an opportunity for dialogue helped create a conversational tone and provide the opportunity to build rapport in the shared project of carrying out the research interview.

Social science literature has shown how rapport, the quality of the personal interaction between interviewer and research participant, makes depth, disclosure, and trust possible. Throughout the training session and debriefing moments, it became apparent that rapport building had to be understood as a process.^27^ Beyond the act of introducing the project and gaining consent in a responsible yet conversational manner, rapport-building was woven throughout the interview. When done well, interviewers introduced themselves warmly and clearly, used accessible language, and checked for comfort early on. They transitioned smoothly into deeper topics, followed the participant’s lead, and listened actively with open body language and minimal interruption.^4^ They responded to emotion with patience and validation, probed gently using participant language, and remained attuned throughout.^28^ At the closing, researchers offered genuine appreciation, summarized key themes, and checked how the participant was feeling—leaving space for final thoughts and ending on a respectful note.^5^ Interestingly, the research team recognized the need to pay greater attention to the physical layout of the interview—from the arrangement of chairs to the availability of an appropriate surface for signing the consent form—as a factor in rapport-building that had remained largely unexamined until that point. Trainees recognized concrete steps of the interview (preparation, introduction, gaining consent) as an opportunity for practice that could allow them to feel more relaxed and less nervous as the interview began.

A final outcome of the training was the recognition that the project’s interview guide required revision to better balance comprehensive coverage with conversational flexibility. Through peer-to-peer feedback, trainees observed that an effective guide should be responsive to the identity and context of each research participant, while also accommodating the interviewer’s own style and needs. Developing this adaptability is a key marker of qualitative literacy: the interview guide should serve as a flexible tool that supports the natural flow of conversation and allows for meaningful probing at appropriate moments of the interview.^29^ This adaptability has important implications for the quality and depth of the data generated, as it enables richer, more contextually grounded responses than rigidly structured questioning alone.

As alluded above, some of these learnings take on a different significance when training novices to interview older adults, particularly those experiencing cognitive decline, as this requires a distinct set of relational and interpretive skills. Older adults living with cognitive decline may offer narratives that often unfold nonlinearly—demanding that interviewers tolerate ambiguity and let participants determine the pace and structure of the conversation. This feature invariably takes more time and presses interviewers to flexibly adapt the interview guide to redirect dialogue.^30^ In this context, the co-construction of meaning inherent in qualitative interviewing becomes especially salient, as interviewers support older adults in articulating experiences that are not only emotionally and cognitively complex, but also sometimes partially beyond their ability to recall. The use of techniques such as reminiscence, as well as other techniques by interviewers can help support verbal communication.

As we learned during team debrief sessions, the challenges of interviewing people with cognitive decline extend beyond the interview itself. Before the conversation begins, researchers must assess the prospective participant’s capacity to consent—determining whether it is ethically appropriate to invite the older adult to take part in research.^31^ After the interview ends, the interviewer must also create space for debriefing and emotional closure, ensuring that participation does not become a source of distress or harm for people recounting experiences that are often suffused with emotion.^27^ The interviewer’s own age, expertise, and social positioning further shape rapport and the flow of disclosure. Simulation training offered team members a valuable opportunity to reflect on these dynamics and begin cultivating ethical and ethnographic sensibilities essential to effectively conduct qualitative interviews with older participants deemed vulnerable: those living with cognitive decline and those other looking after them.^32^

## CONCLUSION

By adapting the standardized patient clinical model into a standardized research participant (SRP) approach, we introduced a structured, rigorous, and replicable method for developing qualitative interviewing skills among novice researchers. This approach offered specific scientific contributions, including enhanced attention to rapport-building, an intentional and flexible approach to study introductions including informed consent, and improved ability to adapt interview guides to both participant context and interviewer style for richer data collection. These features reflect important dimensions of qualitative rigor and support more consistent, reflective interviewing practices with older adults as well as other demographics.

This training was developed in response to specific constraints: the COVID-19 pandemic made traditional pilot interviews difficult, particularly with the vulnerable populations this project aimed to engage. In this context, the SRP model provided a timely and innovative alternative to conventional training methods. While some of the outcomes could have been achieved through classroom-based role-plays or field-based pilot interviews, the simulation-based training offered unique benefits—particularly the opportunity to practice with realistic cases in a low-risk controlled environment and engage in structured feedback. This was especially relevant since our study was about to recruit older adults living with cognitive decline –a vulnerable population

The training was conducted at the UCSF Kanbar Center for Simulation and Clinical Skills, a facility that includes multiple interview rooms equipped with closed-circuit television for live observation and recording. The Kanbar Center also provided access to a pool of trained actors experienced in portraying standardized patients in clinical training. These physical and human resources created an ideal environment for piloting the SRP approach in qualitative research training. However, the economic and logistical demands of simulation-based training may limit its broader adoption, particularly in settings without access to comparable infrastructure.

Nonetheless, we encourage other investigators and research teams—especially those undertaking large-scale qualitative studies or working in academic or nonprofit settings—to consider integrating SRP-based training into their research and capacity-building plans. This may be especially relevant for projects funded by scientific institutions where methodological rigor and replicability are prioritized. Including time and budget for interviewer training at the project start can strengthen both the scientific and ethical foundations of qualitative research. While resource-intensive, the SRP approach offers a scalable and reflective model for preparing researchers to conduct thoughtful, participant-centered interviews.

## Figures and Tables

**Table 1:** Comparing Standardized Patient and Standardized Research Participant (SRP) Case Training Approaches Table 1 compares two simulated training approaches highlighting key features of interest.

Feature	Standardized Patient case	Standardized Research Participant (SRP) case
Training Context	Used in medical or clinical training settings to simulate diagnostic encounters	Used in qualitative research training to simulate semi-structured interviews
Core Aim	To assess clinical reasoning, communication, and diagnostic skills	To build interviewing skills focused on narrative, rapport, and contextual understanding
Content Emphasis	Rich clinical detail: current symptoms, medical history, medications, diagnostic cues	Rich social context: daily routines, caregiving roles, migration histories, relationship dynamics, institutional navigation
Role Adaptation	Actor portrays a specific illness or condition with clinical precision.	Actor may integrate elements of their own lived experience to make portrayal rich and authentic
Feedback Role	Standardized patients evaluate trainees using structured clinical checklists and behavioral rubrics	SRPs provide feedback on interviewer comfort, flow of conversation, and ability to elicit relevant stories
Revisions to Fit SRP Model	Baseline mode	Clinical elements removed or shortened; social and contextual elements emphasized and co-developed with SRPs and simulation team

**Table 2:** Learnings from Research Team Debrief Table 2 summarizes the key learnings from research trainees about different interview stages, including training goals and practical improvement strategies.

Interview Stage	Goal	Improvement Strategy
Entrance	Arrange seating for smooth interview	Assess room layout; pre-arrange furniture; guide SRP to their proposed place, checking for their comfort and ease.
Greeting	Establish rapport with older adults	Structured personal introduction orients SRP to interviewer’s personal identity and role, the research project is presented as a “conversation about your experience” rather than reading a consent form.
Project Introduction	Communicate reason for interview	Well-rehearsed project introduction orients SRP to their role, their value in the project, and what to expect during the interview
Informed Consent	Assess capacity to consent. Complete it while maintaining rapport	Rehearse consent guidance; arrange consent materials for smooth process; anticipate logistics for SRP to sign document;
Recording & gift cards	Handle recorders & gift cards while maintaining rapport	Pre-arrange placement and starting of recorders; rehearse affirmation of recording consent; pre-arrange gift cards
Body of the interview	Cover topics with appropriate degree of emotional attachment and sustain natural conversation flow	Revise interview guide according to personal preferences; practice probes; practice guiding SRP to topics of interest; practice note taking. Use validation, silence and consolation while discussing emotionally charged topics.
Closing interview	Inconspicuously track time; close interview smoothly and allow emotional closure.	Pre-arrange placement of phone or clock; assess need to curtail interview. Leave space and time for Anal thoughts or reflections.

## Data Availability

The datasets used and/or analyzed during the current study are available from the corresponding author on reasonable request.
